# The human papillomavirus oncoproteins: a review of the host pathways targeted on the road to transformation

**DOI:** 10.1099/jgv.0.001540

**Published:** 2021-01-11

**Authors:** James A. Scarth, Molly R. Patterson, Ethan L. Morgan, Andrew Macdonald

**Affiliations:** ^1^​ School of Molecular and Cellular Biology, Faculty of Biological Sciences, University of Leeds, Leeds, West Yorkshire, LS2 9JT, UK; ^2^​ Astbury Centre for Structural Molecular Biology, University of Leeds, Leeds, West Yorkshire, LS2 9JT, UK; ^†^​ Present address: Tumour Biology Section, Head and Neck Surgery Branch, National Institute on Deafness and Other Communication Disorders, National Institute of Health, Bethesda, MD 20892, USA

**Keywords:** cancer, HPV, keratinocyte, oncoprotein, signalling

## Abstract

Persistent infection with high-risk human papillomaviruses (HR-HPVs) is the causal factor in over 99 % of cervical cancer cases, and a significant proportion of oropharyngeal and anogenital cancers. The key drivers of HPV-mediated transformation are the oncoproteins E5, E6 and E7. Together, they act to prolong cell-cycle progression, delay differentiation and inhibit apoptosis in the host keratinocyte cell in order to generate an environment permissive for viral replication. The oncoproteins also have key roles in mediating evasion of the host immune response, enabling infection to persist. Moreover, prolonged infection within the cellular environment established by the HR-HPV oncoproteins can lead to the acquisition of host genetic mutations, eventually culminating in transformation to malignancy. In this review, we outline the many ways in which the HR-HPV oncoproteins manipulate the host cellular environment, focusing on how these activities can contribute to carcinogenesis.

## Introduction

Papillomaviruses are small non-enveloped icosahedral viruses, possessing a circular double-stranded DNA (dsDNA) genome of approximately 8 kb in length [1]. To date, over 400 isolates from a wide variety of fish, reptiles, birds and mammals have been reported in the *Papillomaviridae* family, over 200 of which infect humans [2, 3].

The study of human papillomaviruses (HPVs) has largely been driven by the severity of HPV-associated pathologies: whilst the HPVs that infect the cutaneous epithelium and typically result in benign wart or verruca formation, as well as the low-risk mucosal HPV types 6 and 11 associated with genital warts, have received some attention, the greatest focus has been on the high-risk HPVs (HR-HPVs) associated with the development of cancer [1, 4]. This link between HPV infection and cancer was first established over 35 years ago when HPV16 DNA was found to be present in a large proportion of cervical cancer biopsies [5]. However, it is important to consider that the majority of HR-HPV infections will not progress to cancer; indeed 85 % of cases are subclinical transient infections [6]. There are currently 15 recognized HR-HPV types: HPV16, 18, 31, 33, 35, 39, 45, 51, 52, 56, 58, 59, 68, 73, 82 [7]. Together, HR-HPVs are responsible for >99.7  % of cervical cancer cases, of which 55 % are HPV16-positive and 15 % are HPV18-positive, along with a growing number or oropharyngeal cancers [8]. Cancer of the cervix is the fourth most common malignancy in women worldwide and the fourth leading cause of cancer-related deaths in women: in 2018, around 570 000 people were diagnosed with cervical cancer and over 300 000 deaths worldwide could be attributed to the disease [9]. Furthermore, HR-HPV infection is associated with cancers at a variety of other anogenital sites: around 50% of penile, 25 % of vulvar, 80 % of vaginal and close to 90 % of anal cancers are HPV-driven [10].

Although their expression is insufficient for progression to cancer, the main drivers of HPV-associated pathologies are the oncoproteins E5, E6 and E7 [11]. Together, they act to prolong the proliferation, and delay differentiation, of host keratinocytes to provide an environment suitable for viral replication. Many of the mechanisms by which this is achieved have been widely studied: HR-HPV E7 drives S-phase re-entry via binding to and inducing degradation of the retinoblastoma protein (pRb) and the related pocket proteins p107 and p130 [12–16], whilst E6 concurrently targets p53 for proteasome-mediated degradation, inhibiting pro-apoptotic signalling in response to DNA damage caused by the abnormal S-phase entry [17, 18]. Additionally, E6 modulates a multitude of host signalling pathways, including the Hippo and JAK/STAT pathways, to further promote proliferation and delay differentiation [19–21]. Whilst the role of HPV E5 is not well understood, it has been shown to drive cell proliferation by promoting epidermal growth factor receptor (EGFR)-induced signalling [22–24]. Importantly, if the expression of the viral oncoproteins becomes dysregulated, such as in the context of a persistent infection, cancer progression becomes more likely [11].

In this review, as well as outlining the basic biology of HPV and its life cycle, we aim to provide a comprehensive overview of the biological activities of each HPV oncoprotein in turn, with a particular focus on those of the HR-HPVs. We will illustrate the vital contributions each oncoprotein makes to the virus life cycle as well as addressing how these various functions may contribute towards carcinogenesis.

## Human papillomaviruses

### HPV genome organization

**Fig. 1.**
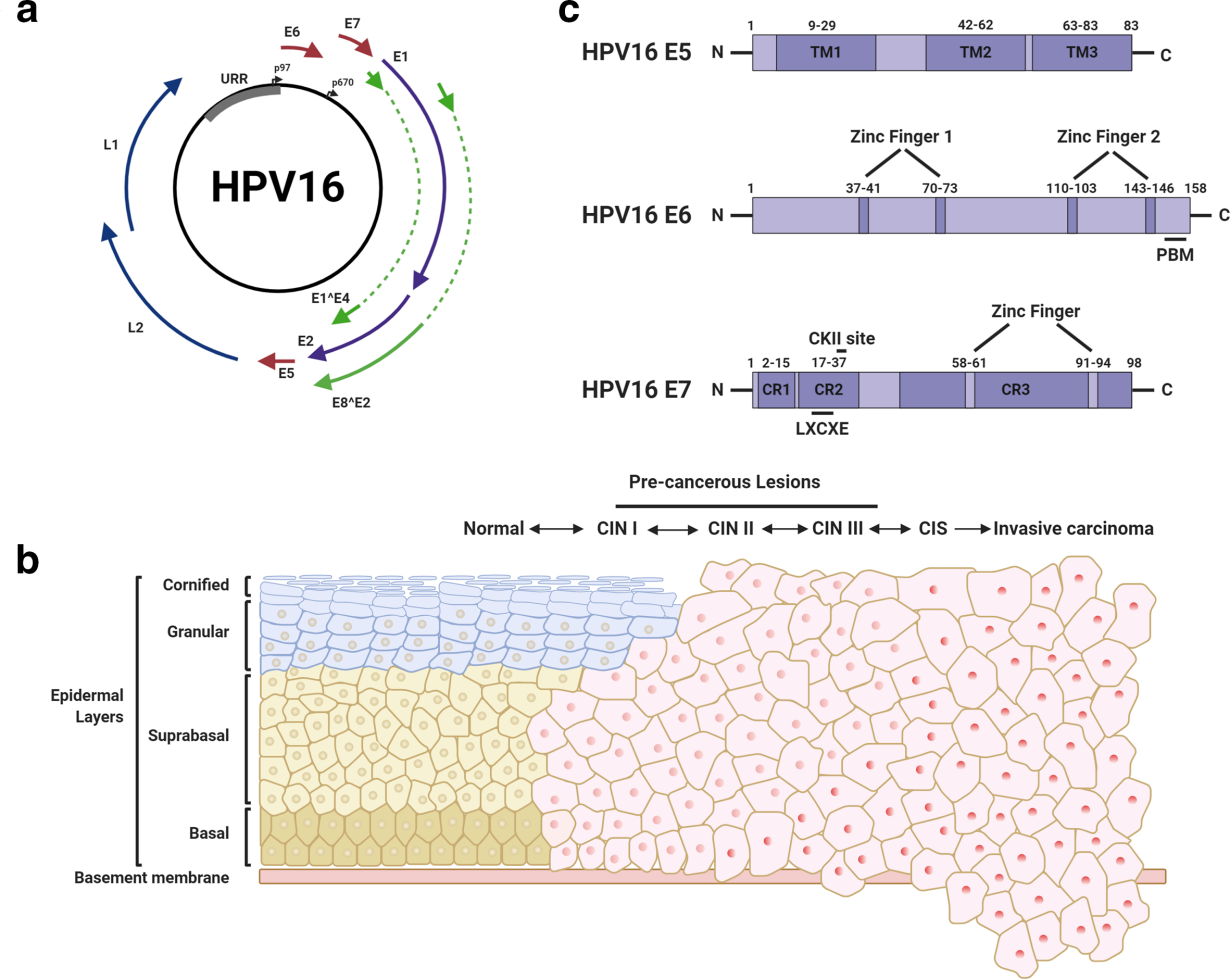
Dysregulation of the HPV life cycle can contribute to carcinogenesis. (a) HPV16 genome organization, displaying the position of viral transcripts. The URR, and early and late promoters are also highlighted. (b) Schematic representation of the development of cervical cancer. Cancer typically progresses from cervical intraepithelial neoplasia (CIN). CIN is classified using a three-stage system based on the proportion of the epithelium displaying abnormalities. CIN1 represents a transient HPV infection, whilst CIN2 and 3 represent a persistent HPV infection with a risk of progression to cancer. Epithelial layers are outlined on the left. CIS, carcinoma *in situ*. (c) HPV16 oncoprotein structure. Numbers refer to amino acid positions. TM, transmembrane domain; PBM, PDZ-binding motif; CR, conserved region; CKII, casein kinase II. Figure created using BioRENDER.com.

The HPV genome is organized into three functional sections: the early (E) region, the late (L) region, and an upstream regulatory region (URR) (Fig. 1a) [8]. Together, the early and late regions consist of nine ORFs, six in the early region (E1, E2, E1^E4, E5, E6, E7 and E8) and two in the late region (L1 and L2), whilst the URR contains the origin of DNA replication (*ori*) as well as the transcription-factor binding sites required for the regulation of RNA-polymerase-II-dependent transcription. The three regions are separated by early and late polyadenylation sites (PAE and PAL, respectively). The HPV genome contains two major promoters: p97 in HPV16 and 31 (p105 in HPV18) is responsible for expression of the early genes and lies upstream of the E6 ORF, and p670 in HPV16 (p811 in HPV18 and p742 in HPV31) is responsible for differentiation-dependent late gene expression and lies within the E7 ORF [25–27]. Although there are multiple minor promoters, their functions are poorly characterized [27]. Importantly, the HR-HPV E6 and E7 oncoproteins are encoded by a single bicistronic mRNA transcript with splicing allowing for the expression of each ORF [27].

### HPV life cycle

The life cycle of HPV is intrinsically linked to the epithelial differentiation programme. For an infection to be established, virions must gain access to the basal lamina of the stratified epithelium via microlesions or by entering cells at the squamo-columnar junction [1, 28]. Primary attachment of virions involves binding of the L1 capsid protein to heparin sulphate proteoglycans (HSPGs) on the basement membrane or on the surface of basal keratinocytes [29]. This induces conformational changes in the capsid structure, resulting in the exposure of the minor capsid protein L2 and binding to secondary receptors, the identity of which remains controversial (see [30] for a review). This is followed by endocytosis, which occurs via a clathrin-, caveolin-, cholesterol-, lipid raft- and dynamin-independent mechanism similar to micropinocytosis [31].

Capsid disassembly occurs in the late endosome, followed by trafficking of the viral genome, in complex with L1 and L2, towards the *trans*-Golgi network (TGN) [32–34]. This is mediated by direct interactions between L2 and components of the retrograde transport machinery such as the retromer complex, an ability conferred by the C-terminal cell-penetrating peptide possessed by L2 [35–37]. Onward nuclear trafficking is critically dependent upon the breakdown of the nuclear envelope during mitosis and requires binding of viral genomes to host mitotic chromosomes via L2 [38–40].

Following nuclear entry, the genome is thought to be rapidly amplified to around 50–100 copies per cell [8]. As HPV does not encode a polymerase, this is achieved via co-option of the host DNA replication machinery, a process mediated by the viral E1 and E2 proteins [41, 42]. Episomal copy number is then maintained in basal cells, where virus protein expression remains minimal due to E2-mediated repression of the p97/p105 early promoter, aiding in avoidance of the immune response [43]. As the infected basal cell divides, one daughter cell remains in the basal layer and acts as an episomal reservoir whilst the other begins to migrate up through the epithelial layers. Here, expression of the viral oncoproteins E5, E6 and E7 delays terminal differentiation and prevents cell-cycle exit in order to provide a suitable environment for further genome amplification, which occurs in an extended G2-like environment [44, 45]. As the oncoproteins do not possess any intrinsic enzymatic activity, this must be achieved by interacting with and modulating the activity of host cellular factors.

The life cycle is completed in the uppermost layers of the epithelium after terminal differentiation has occurred and requires expression of the viral L1, L2 and E1^E4 proteins. Virion assembly occurs in the nucleus after importation of the L1 and L2 capsid proteins, followed by release of newly formed virus particles from the epithelial surface [46].

### Dysregulation of the HPV life cycle during transformation

As is clear from the above section, carcinogenesis is not the default result of the HPV life cycle. Rather, it can be a consequence of a non-productive infection, and represents a ‘dead-end’ for the virus, as despite the synthesis of viral proteins, progeny virions are not produced. In most cases, HR-HPVs typically only cause a short-lived infection, which is cleared over a period of a few months; however in the event of a persistent infection, where the host immune system fails to detect and clear the virus efficiently, transformation may take place (Fig. 1b) [8].

The most important factor required for cancer progression is dysregulation of viral oncoprotein expression, prolonging their pro-proliferative effects upon the host cell [11]. Increased oncoprotein expression is often associated with integration of viral episomes into the host genome and can confer a selective growth advantage to cells [47, 48]. Indeed, the majority of HPV16-associated cervical cancer cases and almost all HPV18-associated cases contain an integrated genome, as do 70 % of HPV-positive (HPV+) head and neck squamous cell carcinoma (HNSCC) cases [49, 50]. Frequently, oncoprotein overexpression is due to disruption of the E2 ORF during integration, preventing E2-mediated repression of E6 and E7 expression [43, 48]. This can also be achieved through methylation of E2 binding sites within the viral URR [51]. Further, integration often results in hybrid virus-host transcripts, which are more stable than viral transcripts, thus potentially enhancing oncoprotein expression [52].

Significantly, given that the majority of HR-HPV infected cells do not progress to cancer, oncoprotein expression is necessary yet not sufficient for transformation [6]. It follows, therefore, that other genetic insults to host oncogenes and tumour suppressors are also required for progression to occur [49, 50]. Despite this, the cellular environment created by the activities of the HR-HPV oncoproteins discussed in the following sections of this review – that of rapid cell-cycle progression, abrogated cell-cycle checkpoints and genomic instability – is highly conducive to the acquisition of such host genetic aberrations.

## The E5 oncoprotein

The E5 protein is a poorly understood viral protein of between 8 and 9.5 kDa expressed by a subset of HPV types, including the high-risk HPV16, 18 and 31 [53]. E5 is a membrane integrated, highly hydrophobic protein and HPV16 and 18 E5 proteins contain three putative α-helical structures that function as transmembrane domains (TMDs) (Fig. 1c). The lack of antibody reagents for HPV E5 has hampered investigations into its biological functions during infection [53]; however, overexpression studies have elucidated some important aspects of E5 biology. HPV E5 primarily localizes to the endoplasmic reticulum and the Golgi apparatus, with a small proportion also observed at the perinuclear region and plasma membrane [54]. Furthermore, HPV16 E5 has been shown to oligomerize both *in vitro* and in cells [55]; however, this has not been observed in HPV-infected cells due to the lack of suitable reagents discussed above.

As an early gene, E5 plays an important role during the productive viral life cycle. Using HPV genomes lacking a functional E5 ORF, studies indicate that HPV16, HPV18 and HPV31 E5 proteins have no apparent role in regulating viral genome maintenance or the proliferative ability of undifferentiated keratinocytes [56–58]. In contrast, E5 has a clear role in the differentiation-dependent stages of the HPV life cycle. Our group demonstrated that HPV18 E5 is required for unscheduled DNA synthesis in suprabasal cells, but not for genome amplification or the expression of late viral proteins [56]. Interestingly, defective expression of the late viral protein E1^E4 was reported for HPV31 E5 knock out (E5KO) genomes, yet no E1^E4 defect has been observed with the corresponding HPV16 and HPV18 E5KO genomes [56–58]. These data therefore suggest that E5 proteins from different HPV types may have distinct roles during the productive viral life cycle.

The major transforming proteins of HPV are E6 and E7; however, for bovine papillomavirus (BPV) type 1, the major transforming protein appears to be E5 [59]. One critical function of BPV1 E5 is activation of the platelet-derived growth factor receptor (PDGFR), resulting in downstream mitogenic signalling, enhanced DNA synthesis and transformation [60, 61]. This occurs via direct binding of BPV1 E5 to the PDGFRβ isoform [62]. In contrast, there is no evidence for BPV4 E5 activation of growth-factor receptors; rather it disrupts the expression and activity of key cell-cycle regulators such as cyclin A and p27^KIP1^ [63]. Significantly, HPV E5 also does not bind to or activate the PDGFR, and HPV E5 proteins alone were found to be weakly oncogenic in comparison to HPV E6 and E7 in transgenic mouse models where the oncoproteins were expressed under the control of the epithelial specific keratin-14 (K14) promoter [64]. Furthermore, the expression of E5 is not detected in all HPV+ tumours, suggesting its contribution to transformation may rather be in the modulation or enhancement of E6 and E7 functions [65–67]. Indeed, E5 was demonstrated to enhance the oncogenic abilities of HPV16 E7 in primary baby-rat kidney cells [66], whilst in K14 transgenic mouse models, tumour formation was greater in mice expressing HPV16 E5/E6 or E5/E7, when compared with E6 or E7 alone [67]. Interestingly, tumour formation in mice expressing E5 alone was only observed after treatment with oestrogen, an important driver of cervical carcinogenesis, indicating that E5 may promote, rather than initiate, tumourigenesis [67].

**Fig. 2.**
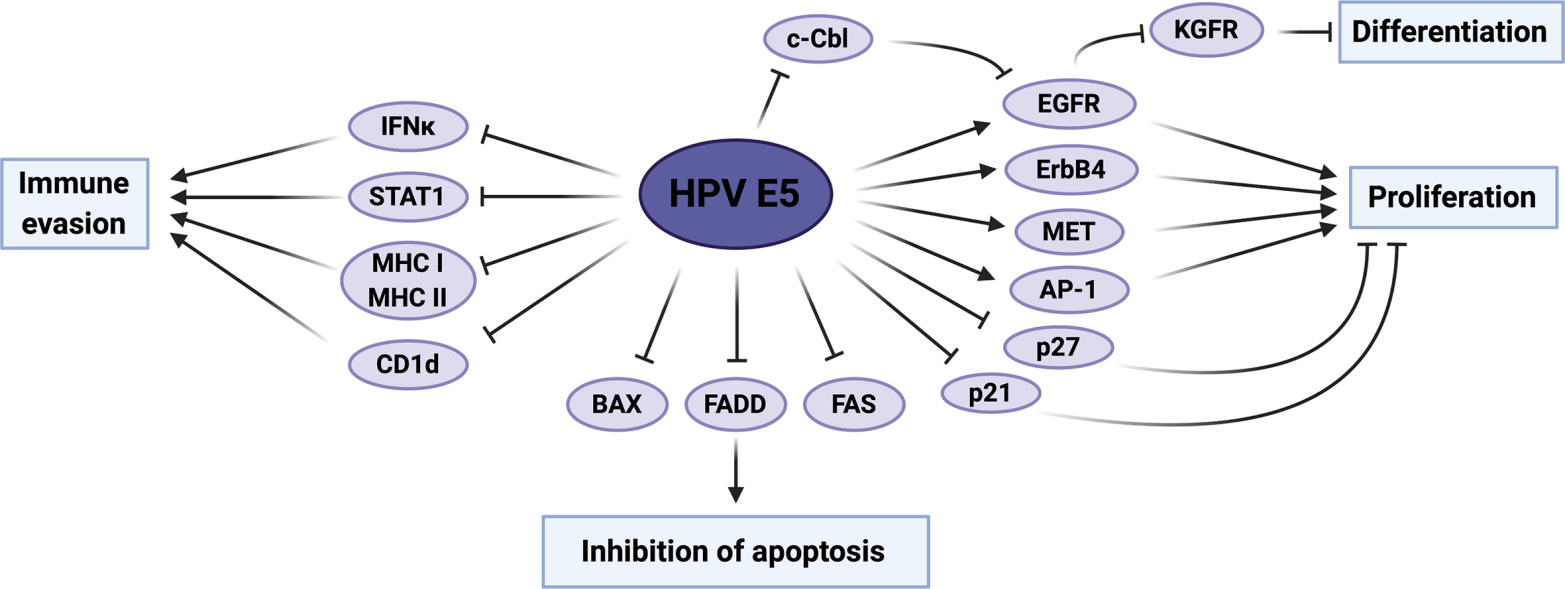
Summary of HPV E5 activities which contribute towards host-cell transformation. The host proteins and pathways reported to be modulated by HPV E5 are displayed, as well as the functional impact these have on the host cell. STAT, signal transducer and activator of transcription; MHC, major histocompatibility complex; EGFR, epidermal growth factor receptor; KGFR, keratinocyte growth factor receptor; AP-1, activator protein 1; FADD, Fas-associated protein with death domain. Figure created using BioRENDER.com.

These studies have prompted investigations into how HPV E5 contributes to oncogenesis (Fig. 2). Many studies have demonstrated that E5 binds to a range of host proteins, several of which have been shown to function in the dysregulation of critical biological pathways associated with cellular transformation (Table 1).

**Table 1.** Host proteins known to interact with HPV E5

Protein name	HR-HPV	LR-HPV	Proposed function	Reference
16K subunit of v-ATPase	+	+	Endosomal acidification	[54, 275]
A4	+	?	Increased proliferation	[95]
Bap31	+	?	Increased proliferation	[96]
Calnexin	+	?	Immune evasion	[88]
Calpactin I	+	?	Induction of koilocytosis	[276]
EGFR	+	?	Increased proliferation	[74]
ErbB4	+	?	Increased proliferation	[68]
EVER1	+	?	Promotes AP-1 activation	[277]
EVER2	+	?	Promotes AP-1 activation	[277]
HLA-I heavy chain	+	?	Immune evasion	[94]
Karyopherin β3	+	?	Unknown	[278]
YIPF4	+	+	Unknown	[279]
ZnT-1	+	?	Promotes AP-1 activation	[277]

+, interaction confirmed; ?, binding ability not analysed.

### Induction of cell proliferation

Early studies suggested that, similar to the BPV1 E5 protein, one of the primary oncogenic functions of HPV E5 is the modulation of growth-factor signalling [22, 23]. To this end, HPV E5 has been shown to bind the growth-factor receptor ErbB4, promoting cell proliferation [68]. However, E5-mediated EGFR activation has been the most extensively studied aspect of its biological functions [22–24]. Our group demonstrated that HPV18 E5-induced EGFR signalling is critical in maintaining unscheduled DNA synthesis and cell-cycle progression during keratinocyte differentiation, suggesting that this function of E5 may contribute to both the virus life cycle and E5-mediated tumourigenesis [56]. Interestingly, E5 activation of the EGFR is essential for E5-induced hyperplasia and cellular transformation *in vivo* [69]. Further, recent studies have demonstrated that the activation of EGFR by E5 can also induce the expression of the growth-factor receptor c-Met, a potent oncogene [70].

Several mechanisms for EGFR activation by HPV E5 have been proposed. Initially, the interaction of E5 with the 16K subunit of the vacuolar H^+^-ATPase (v-ATPase) was suggested to abrogate its role in the endosomal acidification process, resulting in reduced EGFR degradation after growth-factor stimulation [71]. However, subsequent studies have shown that this may not be the case [72, 73]. HPV16 E5 has also been shown to bind directly to the EGFR, but this is not thought to be critical for E5-mediated EGFR activation [74]. HPV E5 can also disrupt the interaction between the EGFR and the E3 ligase c-Cbl, reducing EGFR ubiquitination and degradation, thereby enhancing mitogenic signalling [75].

Our lab has demonstrated an additional mechanism of EGFR activation induced by HPV16 E5. As mentioned above, HPV16 E5 forms oligomers and detailed biochemical studies demonstrated that E5 can function as a virus-encoded ion channel, a so-called viroporin [76, 77]. Interestingly, well-characterized broadly acting inhibitors of other viroporins, such as rimantadine, also inhibit E5 viroporin activity resulting in reduced E5-mediated EGFR activation [78]. Furthermore, this function of E5 was also shown to be important for the expression of mediators of cell-cycle progression, suggesting that viroporin activity may play a role in malignant transformation [78]. Further studies are required to identify the exact mechanism of EGFR activation by HPV E5.

As well as enhancing EGFR signalling, HPV16 E5 also downregulates expression of the anti-proliferative keratinocyte growth-factor receptor (KGFR) [56, 79]. Interestingly, E5-mediated downregulation of KGFR, also known as FGFR2b, is associated with an upregulation of its splice-isoform FGFR2c [80]. This isoform is highly expressed in mesenchymal-like cells and the induction of FGFR2c by HPV16 E5 promotes epithelial-mesenchymal transition (EMT), an important event in the progression to malignant transformation [80].

In addition to the modulation of growth-factor receptor signalling, HPV E5 can also promote cell proliferation by other mechanisms. Early studies demonstrated that HPV11 and HPV16 E5 can induce expression of the proto-oncogene c-Jun, a key driver of cell proliferation [81]. Furthermore, whilst HPV16 E5 promoted mitogen-activated protein kinase (MAPK) signalling in response to EGF in the mouse 3T3 fibroblast cell line, it also induced EGF-independent activation of MAPK via protein kinase C (PKC) signalling [82]. HPV E5 can also directly regulate cell-cycle progression via the inhibition of p21^WAF1/CIP1^ and p27^KIP1^ [83, 84].

### Inhibition of apoptosis

An important hallmark of cancer cells is the ability to avoid apoptosis, particularly in response to death ligands. As a virus, HPV induces stress upon infected cells and the abrogation of cell-cycle checkpoints during infection promotes apoptosis. HPV has therefore evolved efficient mechanisms to prevent cell death. HPV16 E5 contributes towards this by promoting the proteasomal degradation of the pro-apoptotic Bcl-2 family member BAX upon reactive oxygen species (ROS)-induced apoptosis [85]. Furthermore, HPV16 E5 can prevent FasL- and TNF-related apoptosis-inducing ligand (TRAIL)-induced apoptosis by downregulating expression of the Fas receptor and inhibiting recruitment of Fas-associated protein with death domain (FADD) to form the death-induced signalling complex (DISC) [86]. HPV16 E5 can also inhibit the apoptotic response to ultraviolet (UV)-B radiation; this requires E5-dependent activation of EGFR and downstream signalling through phosphatidylinositol 3-kinase (PI3K) and extracellular signal-regulated kinase 1/2 (ERK1/2) [87].

### E5-mediated immune evasion

E5 has also been shown to modulate the immune response to infection by interfering with the expression or trafficking of several critical immune receptors, including Major Histocompatibility Complex (MHC) class I, MHC class II and CD1d [88–92]. The cell-mediated arm of the adaptive immune response, which plays an essential role in the detection of virus-infected cells, functions by recognizing foreign protein epitopes on the surface of an infected cell in complex with MHC class I. E5 proteins from several HPV types (2a, 16 and 83) have been shown to specifically downregulate HLA-A and HLA-B, abrogating their cell-surface expression and inhibiting T-cell recognition of HPV-infected cells [89, 90]. HPV16 E5 can bind directly to MHC class I via its first TMD, which may act to retain MHC class I within the Golgi apparatus, preventing its onward trafficking to the cell surface [93, 94]. Other mechanisms, such as the formation of a ternary complex with MHC class I and the chaperone calnexin, and/or a quaternary complex between HPV E5, MHC class I, Bap31 and A4, have also been speculated as possible explanations for the retention of MHC class I in the Golgi [88, 95, 96]. HPV E5 can additionally impede cell-surface expression of MHC class II and CD1d, suggesting that HPV E5 promotes subversion of the immune system via multiple mechanisms [91, 92].

The E5 oncoprotein is also capable of dysregulating interferon (IFN) signalling. HPV16 E5 can suppress the expression of STAT1, thus preventing downstream IFN-stimulated gene (ISG) transcription [97]. Furthermore, E5 silences expression of IFNκ, a key keratinocyte-specific IFN involved in antiviral immunity; although the exact mechanism for this remains to be determined, silencing results in abrogated ISG expression [97]. Interestingly, the effects on IFNκ expression were found to be, at least in part, dependent upon E5-induced EGFR signalling.

Recent data has suggested a potential role for E5 viroporin activity in the modulation of the host immune response. In a mouse model of HNSCC, HPV16 E5 expression resulted in a downregulation of MHC class I and this provided resistance to anti-programmed death-ligand 1 (anti-PD-L1) immunotherapy [98]. However, co-treatment with the viroporin inhibitor rimantadine sensitized tumours to anti-PD-L1 treatment, suggesting that E5 viroporin activity is responsible for the resistance to these therapies and could hence be therapeutically targeted using the currently available inhibitors [98].

## The E6 oncoprotein

E6 was first suspected to be an oncoprotein after analysis of cervical tumours and cervical cancer-derived cell lines revealed that the E6 ORF was retained during viral-genome integration and was highly expressed [99]. Follow-up experiments in human mammary epithelial and rat primary kidney cells confirmed that E6 possessed intrinsic transforming activities [100, 101]. Despite sharing some activities with their high-risk counterparts, low-risk E6 proteins are unable to induce transformation in primary cells [102]. Interestingly, studies have demonstrated that E6 and E7 likely cooperate in inducing tumour formation: E7 drives early carcinogenesis whilst E6 accelerates progression towards malignancy [103]. In support of this, both the E6 and E7 ORFs were found to be required in the context of the full viral genome for the immortalization of primary human keratinocytes [104].

**Fig. 3.**
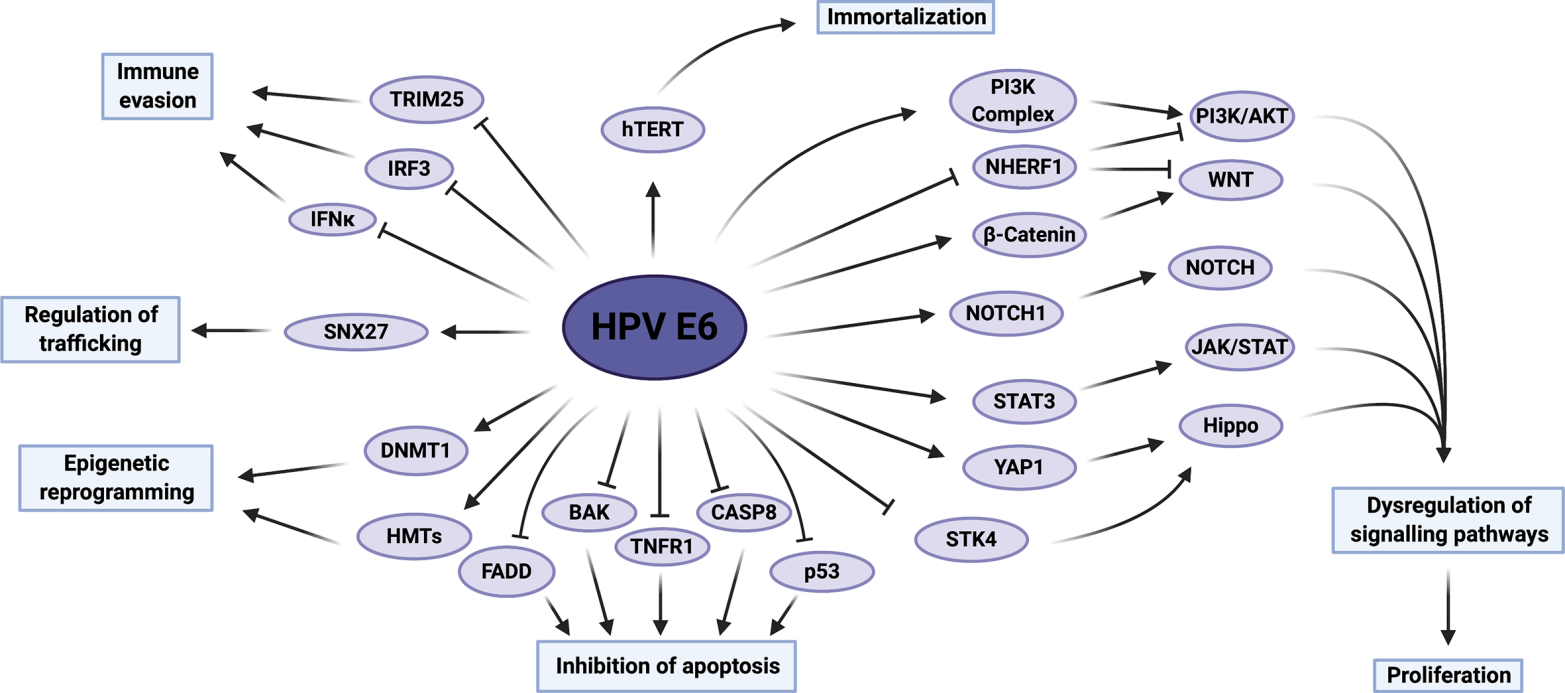
Summary of HPV E6 activities which contribute towards host-cell transformation. The host proteins and pathways reported to be modulated by HPV E6 are displayed, as well as the functional impact these have on the host cell. hTERT, human telomerase reverse transcriptase; PI3K, phosphatidylinositol 3-kinase; NHERF1, Na^+^/H^+^ exchange regulatory factor 1; STAT, signal transducer and activator of transcription; JAK, Janus kinase; YAP1, Yes-associated protein 1; STK4, serine/threonine-protein kinase 4; CASP8, caspase-8; TNFR1, tumour necrosis factor receptor 1; BAK, Bcl-2 homologous antagonist/killer; FADD, Fas-associated protein with death domain; HMTs, histone methyltransferases; DNMT1, DNA methyltransferase 1; SNX27, sorting nexin 27; IRF3, IFN regulatory factor 3; TRIM25, tripartite motif-containing protein 25. Figure created using BioRENDER.com.

E6 proteins are approximately 150 amino acids long and contain two zinc-binding motifs formed by two pairs of CXXC sequences, which are essential for the activities of E6 (Fig. 1c) [105]. Solving of the E6 structure by NMR revealed that the protein has two zinc-finger domains termed E6N and E6C, each formed of a three-stranded β-sheet and two short helices, connected by a short linker helix [106, 107]. Further, high-risk E6 proteins contain a C-terminal PDZ (PSD-95/DLG/ZO-1) binding motif (PBM) formed of an X-S/T-X-V/L sequence (Fig. 1c) [108]. E6 is able to target a multitude of host cellular factors and perturb host signalling pathways despite a lack of intrinsic enzymatic activity; these are summarized in Fig. 3, Table 2.

**Table 2.** Host proteins known to interact with HPV E6

Protein name	HR-HPV	LR-HPV	Proposed function	Reference
14-3-3ζ	+	-	Stabilization of E6	[133]
ADA3	+	-	Inactivation of p53	[113]
AIF	+	+	Increased survival	[280]
BAK	+	+	Increased survival	[115. 116]
BARD1	+	?	Defective DNA repair	[281]
BCCIPβ	+	?	Promotes p53 ubiquitination	[282]
BRCA1	+	?	Defective DNA repair	[283]
CARM1	+	+	Epigenetic reprogramming	[175]
CASK	+	HPV40 only	Unknown	[132, 160]
Caspase-8	+	-	Increased survival	[284]
CBP	+	low	Inactivation of p53	[111, 112]
c-Myc	+	-	Host-cell immortalization	[285]
CYLD	+	?	Increased proliferation	[286]
DLG1	+	HPV40 only	Increased proliferation	[127, 128, 132]
DLG2	+	-	Unknown	[160]
DLG4	+	-	Unknown	[160, 287]
DVL2	+	?	Increased proliferation	[139]
E6-AP	+	+	Degradation of p53	17, 18, 288]
FADD	+	?	Increased survival	[117]
GIPC1	+	?	Inhibition of TGFβ signalling	[289]
GOPC	+	-	Unknown	[290]
GPS2	+	+	Unknown	[291]
HERC2	+	low	Unknown	[292, 293]
hTERT	+	?	Host-cell immortalization	[294]
INADL	+	-	Disruption of cell polarity	[295, 296]
IRF3	+	-	Immune evasion	[166]
KAT5	+	+	Activation of the HPV early promoter	[297]
LIN7C	+	HPV40 only	Unknown	[132, 160]
MAGI1	+	-	Disruption of cell polarity	[125]
MAGI2	+	-	Disruption of cell polarity	[126]
MAGI3	+	-	Disruption of cell polarity	[126]
MCM7	+	low	Chromosomal instability	[298]
MGMT	+	?	Defective DNA repair	[299]
MPDZ	+	-	Increased proliferation	[300]
MPP7	+	HPV40 only	Unknown	[132, 160]
NFX1-123	+	-	Host-cell immortalization	[301, 302]
NFX1-91	+	-	Host-cell immortalization	[301, 302]
NHERF1	+	+	Wnt signalling activation	[141, 154]
p300	+	low	Inactivation of p53	[111, 112]
p53	+	low	Inhibition of apoptosis	[17, 109, 114, 303, 304]
PML	+	+	Inhibition of PML-induced senescence	[305]
PRMT1	+	+	Epigenetic reprogramming	[175]
PTPN13	+	-	Increased proliferation	[306, 307]
PTPN3	+	-	Increased proliferation	[160, 308]
RCN2	+	-	Unknown	[309]
SCRIB	+	-	Disruption of cell polarity; increased E6 expression	[124, 132, 160, 310]
SET7	+	+	Epigenetic reprogramming	[175]
SIPA1L1	+	-	Increased Rap1 GTPase activity	[311, 312]
SNTB2	+	-	Unknown	[[132]
TAX1BP3	+	-	Increased cell motility	[313]
TNFR1	+	?	Increased survival	[119]
TRIM25	+	+	Immune evasion	[168]
TSC2	HPV16 only	-	Increased mTORC1 signalling	[155]
TYK2	+	low	Immune evasion	[167]
USP15	+	+	Immune evasion	[168, 220]
XRCC1	+	-	Defective DNA repair	[314]
ZO-2	+	-	Increased cell migration	[132]
Zyxin	-	HPV6 only	Unknown	[315]

+, interaction confirmed; -, no interaction recorded; low, limited binding detected; ?, binding ability not analysed.

### Blockade of pro-apoptotic signalling

The best-studied function of HR-HPV E6 is the degradation of the p53 tumour suppressor protein in order to disrupt pro-apoptotic signalling [109]. p53 is a sequence-specific DNA binding protein that serves as an effector of DNA damage and is mutated or inactivated in over half of all human cancers [110]. It is capable of inducing cell-cycle arrest, cellular senescence and apoptosis through transcriptional regulation in response to a diverse range of stresses, and is itself under exquisite control by a number of mechanisms, primarily by the E3 ubiquitin ligase mouse double minute 2 homologue (MDM2). Under normal cellular conditions, p53 undergoes rapid turnover due to binding of MDM2 and subsequent ubiquitination-dependent proteasomal degradation. Cellular stress-induced phosphorylation of p53 precludes binding of MDM2, thus permitting transcriptional activation of target genes [110]. HR-HPV E6 can interact with the host E6-associated protein (E6-AP), a HECT domain-containing E3 ubiquitin ligase, by binding to its conserved LXXLL motif [17]. This allows the formation of a ternary complex with p53, which leads to its proteasomal degradation, thereby inhibiting p53-mediated apoptosis [18].

Importantly, E6-induced degradation does not totally deplete the host cell of p53, and low-risk E6 does not demonstrate an ability to degrade p53. HPV E6 proteins have therefore evolved additional degradation-independent mechanisms of inhibiting p53 signalling. E6 can prevent the acetylation of p53 by the related acetyltransferases p300 and CBP (CREB-binding protein) via the formation of a p53-E6-p300/CBP complex [111, 112]. E6 also inhibits the function of ADA3, which similarly promotes p53 acetylation and thus stabilization; although in contrast to p300/CBP, this is achieved via degradation [113]. Direct binding of both high-risk and low-risk E6 proteins to the C-terminus of p53 has also been reported, as well as sequestration of p53 in the cytoplasm; these act to prevent p53 from interacting with DNA [105, 114]. Whilst the primary aim of these activities is to abrogate pro-apoptotic signalling in response to HPV E7-induced hyperproliferation, in the context of a persistent infection the prolonged loss of p53 function allows the accumulation of genetic mutations and thus contributes to cancer progression.

In addition to targeting p53, E6 targets other proteins involved in apoptosis. This includes binding to the host pro-apoptotic protein Bcl-2 homologous antagonist/killer (BAK) in order to suppress intrinsic apoptosis signalling. Similar to p53, E6 complexes with E6-AP to target BAK for degradation [115, 116]. Furthermore, E6 also dysregulates the extrinsic apoptosis pathway, which transmits extracellular apoptotic stimuli from the cell surface, through interactions with FADD and caspase-8, leading to their degradation [117, 118]. As these proteins are required to potentiate signalling from all death receptors, this allows the virus to inhibit extrinsic signalling from multiple receptors at once. HPV16 E6 has also been shown to directly bind the C-terminus of the transmembrane death receptor tumour necrosis factor receptor 1 (TNFR1), further disrupting pro-apoptotic signalling [119].

### Interaction with PDZ proteins

A property unique to the E6 oncoproteins of HR-HPVs is the presence of a C-terminal PBM [120]. This motif permits binding to a range of host proteins which possess PDZ domains, a protein–protein interaction motif of ~90 amino acids in length. An interaction with E6 has been confirmed for at least 19 PDZ proteins to date, although this represents only a fraction of the ~200 host PDZ proteins [108]. Despite this, the range of proteins possessing PDZ domains clearly allows E6 to regulate multiple aspects of the host-cell environment. Significantly, p53 degradation-defective HR-HPV E6 proteins are still capable of immortalizing cells, but those defective in binding PDZ partners are unable to induce epithelial hyperplasia, underlining the importance of modulating the activity of host PDZ proteins [121, 122]. Interestingly, several of the PDZ proteins confirmed to bind HPV E6, including SCRIB, discs large homologue 1 (DLG1), Na^+^/H^+^ exchange regulatory factor 1 (NHERF1) and the MAGI family of proteins, are regulators of cell polarity and cell–cell contacts [123–128]. The interaction with E6 results in the proteasomal degradation and/or mislocalization of the PDZ proteins, hence disrupting cell polarity, a common characteristic of malignant cells. E6 also targets sorting nexin 27 (SNX27), utilizing the PDZ domain to dysregulate trafficking of proteins such as glucose transporter 1 (GLUT1), essential for glucose uptake, thus disrupting cellular homeostasis and proliferation [129]. Furthermore, our lab recently demonstrated that HR-HPV E6 proteins promote host cell proliferation and survival in a PBM-dependent manner via the activation of c-Jun N-terminal kinase 1/2 (JNK1/2) [130]. Taken together, these observations have led to the hypothesis that targeting of PDZ proteins contributes significantly to HPV-induced transformation [131]. Indeed, the number of PDZ proteins bound by E6 correlates strikingly with the potential for oncogenicity: although binding to DLG1 is shared by all HR-HPV E6 proteins, only those of HPV16 and 18 are capable of binding SCRIB [132]. Binding of E6 to PDZ proteins can be abrogated by phosphorylation of a threonine residue within the PBM of E6 by AKT in the case of HPV16, or, for HPV18, by PKA [133].

### Host-cell immortalization

E6 can promote oncogenesis through host-cell immortalization due to activation of the telomerase complex [134]. Increased telomerase activity and the subsequent lengthening of telomeres permits indefinite proliferation by circumventing the onset of senescence associated with excessive telomere shortening. Expression of HR-HPV E6 is necessary and sufficient for telomerase activation: this is achieved via upregulated expression of the catalytic subunit of telomerase, human telomerase reverse transcriptase (hTERT) [135, 136]. Significantly, low-risk E6 proteins do not activate telomerase [137]. The mechanisms by which E6 promotes hTERT expression are complex and multi-layered (see [134] for a review): it hijacks several host factors, including specificity protein 1 (Sp1) and c-Myc to activate transcription, whilst also modulating the transcriptional repressors upstream stimulating factor 1 and 2 (USF1 and USF2).

### Dysregulation of host signalling networks

**Fig. 4.**
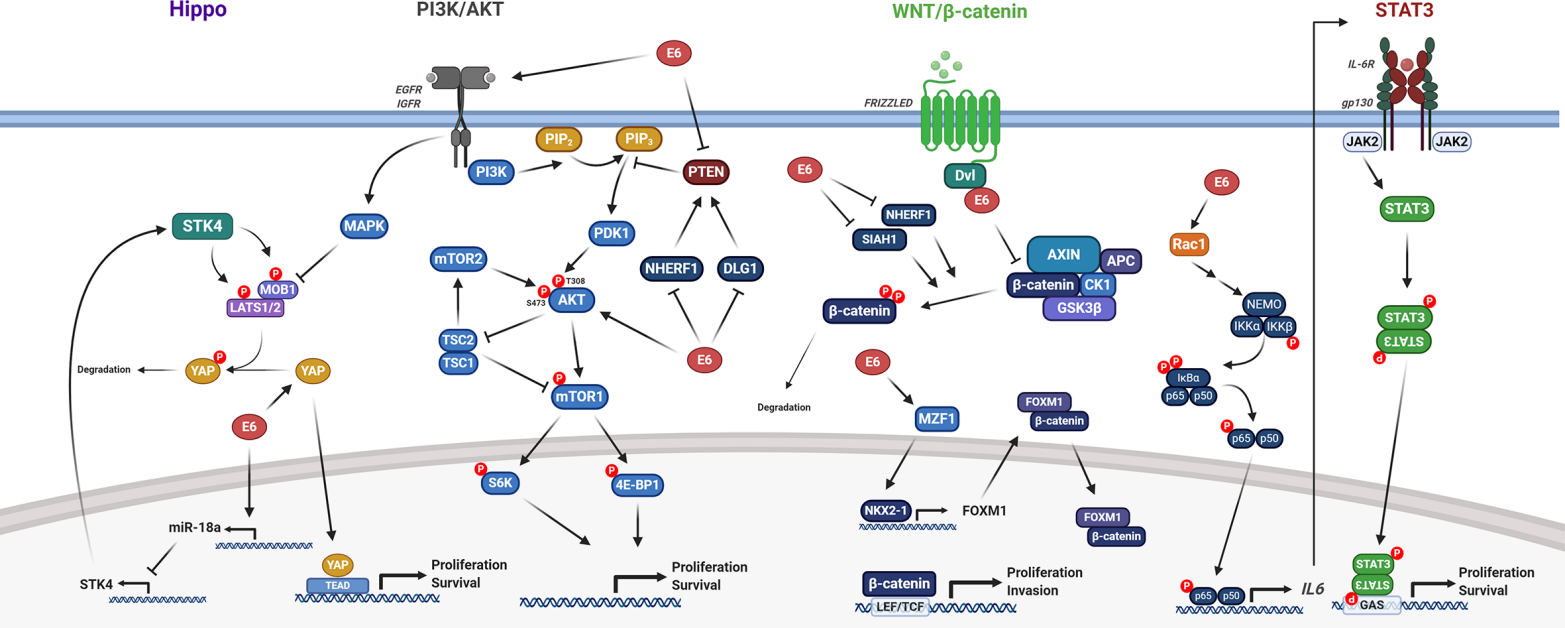
HPV E6 modulation of host signalling pathways. Summary of the mechanisms by which HR-HPV E6 dysregulates the host Hippo, PI3K/AKT, Wnt/β-catenin and JAK/STAT signalling pathways. See text for details. Figure created using BioRENDER.com.

E6 achieves many of its oncogenic functions by dysregulating a multitude of host signalling pathways (Fig. 4). Collectively, this acts to drive proliferation of host cells, typically by modulating the expression of downstream target genes associated with cell growth and survival. The pathways reported to be hijacked by E6 are outlined in turn in this section.

#### Wnt signalling pathway

The Wnt pathway is a highly conserved signalling axis implicated in the regulation of development, but is also commonly dysregulated in cancer [138]. The main effector of canonical Wnt signalling is β-catenin which, in the absence of Wnt ligands, is rapidly phosphorylated by glycogen synthase kinase 3 beta (GSK3β), targeting it for ubiquitin-mediated degradation. Conversely, binding of a Wnt ligand to its cognate receptor leads to the stabilization and nuclear accumulation of β-catenin and the transcription of genes associated with proliferation [138]. HPV16 E6 can bind directly to Dishevelled 2 (DVL2), a protein which impedes formation of the β-catenin degradation complex; this promotes β-catenin nuclear accumulation [139]. Additionally, evidence indicates that the E6/E6-AP complex can further stabilize β-catenin [140]; subsequent studies indicate this is likely associated with the ability of E6/E6-AP to interact with and induce the proteasomal degradation of NHERF1, a negative regulator of canonical Wnt signalling [141]. E6 is also reported to upregulate forkhead box protein M1 (FOXM1) expression, a β-catenin binding partner, which promotes its nuclear localization, via a myeloid zinc finger 1 (MZF1)/NK2 homeobox 1 (NKX2-1) signalling axis [142]. Further, cells in which expression of E6 and E7 is repressed display enhanced seven in absentia homologue 1 (SIAH1) expression, a protein implicated in β-catenin degradation, suggesting the oncoproteins may, to some degree, cooperate in dysregulating the Wnt pathway [143]. Unconstrained activation of the Wnt pathway leads to uncontrolled promotion of biological processes such as proliferation, differentiation and tumourigenesis.

#### Notch signalling pathway

The Notch pathway is a highly conserved signalling pathway essential for the regulation of proliferation, cell fate and differentiation [144]. The pathway is activated upon Notch ligand-receptor binding at the surface of adjacent cells, which induces a cascade of cleavage events to release the Notch intracellular domain (NICD) from the membrane-bound receptor. NICD translocates to the nucleus to stimulate transcription of target genes in concert with the coactivator Mastermind-like protein 1 (MAML1). Aberrant activation of Notch has been reported in a wide variety of cancers, and intriguingly both tumour suppressive and oncogenic roles for the pathway have been proposed, but the role of Notch signalling during HR-HPV infection and cervical cancer is less clear [145]. The Notch1 receptor has been observed to be transcriptionally upregulated in HPV16 E6-containing keratinocytes and also to increase with cervical cancer progression [146, 147]. Additionally, expression of both Notch receptors and ligands is upregulated in a significant proportion of HNSCC cases [148]. Conversely, a different study identified specific downregulation of the Notch1 receptor in HPV+ cervical cancer cell lines and invasive cervical carcinoma biopsies, and showed that reintroduction of NCID abrogated HPV oncoprotein expression [149]. Clearly, therefore, much remains to be understood regarding the role of Notch signalling in HPV-driven carcinogenesis.

#### PI3K/AKT/mTOR signalling pathway

The PI3K/AKT/mTOR signalling pathway is a key regulator of proliferation, metabolism and motility, and one of the most frequently dysregulated pathways in human cancers [150]. Given this essential role in the regulation of proliferation, it is unsurprising that HPV E6 dysregulates the pathway at multiple points. A key stage in this signalling axis involves the activation of class I PI3Ks by receptor tyrosine kinases (RTKs), allowing the phosphorylation of phosphatidylinositol 4,5-bisphosphate (PIP2) to generate phosphatidylinositol 3,4,5-trisphosphate (PIP3), a short-lived second messenger. This can be reversed, and the pathway thereby inactivated, through the phosphatase and tensin homolog (PTEN), a lipid phosphatase and *bona fide* tumour suppressor. Early reports demonstrated that HPV18 E6 expression was sufficient to induce increased PI3K and AKT phosphorylation [151]. This was hypothesized to be, at least in part, associated with the ability of HPV E6 to bind and degrade the PDZ domain-containing protein DLG1, a binding partner of PTEN, which promotes its stabilization [151, 152]. Furthermore, NHERF1, in addition to its role as a negative regulator of the Wnt pathway, can also inhibit PI3K signalling by promoting the recruitment of PTEN [153]. Therefore, E6-induced degradation of NHERF1 also contributes to uncontrolled PI3K/AKT signalling [154].

One of the many downstream targets of AKT is the mammalian target of rapamycin complex 1 (mTORC1). mTORC1 kinase activity is indirectly inhibited via the tuberous sclerosis complexes 1/2 (TSC1/2), which in turn are negatively regulated by AKT phosphorylation [150]. It was initially thought mTORC1 could be activated by HPV16 E6-induced degradation of TSC2 in an E6-AP-dependent manner [155, 156]. However, subsequent studies cast doubt upon these findings as no evidence for TSC2 degradation was apparent; rather mTORC1 activation was concluded to be achieved in an AKT-dependent manner [157]. Later, hyperactivity of a range of RTKs, including the EGFR, the insulin receptor (INSR) and the insulin-like growth factor receptor (IGF1R), was confirmed to contribute towards PI3K/AKT/mTOR pathway activation in HPV16 E6-expressing foreskin keratinocytes [158].

#### Hippo signalling pathway

The Hippo signalling pathway is a key pathway in organ homeostasis and is dysregulated in many types of cancer [159]. Activation of the canonical Hippo pathway results in the induction of a kinase cascade, ultimately leading to the phosphorylation of the downstream transcriptional regulators Yes-associated protein 1 (YAP1) and transcriptional coactivator with PDZ-binding motif (TAZ). This promotes binding of 14-3-3 proteins to YAP1/TAZ and hence their cytoplasmic retention, ultimately resulting in proteasomal degradation. However, when the Hippo pathway is inactive, YAP1/TAZ localize to the nucleus and interact with the TEA domain (TEAD) family of transcription factors, leading to transcription of genes involved in cell proliferation and survival.

HR-HPV is capable of dysregulating the Hippo signalling pathway at several stages. E6 has been shown to upregulate YAP1 protein levels by shielding it from proteasomal degradation in a manner dependent on its PBM, resulting in the nuclear accumulation of YAP1 in both cervical tumours and HPV-containing keratinocytes [19, 160]. Indeed, YAP1 overexpression alone is sufficient to induce cervical dysplasia [161]. Additionally, our studies have demonstrated that expression of the serine/threonine-protein kinase 4 (STK4), a key upstream negative regulator of YAP1/TAZ activity, is significantly downregulated in HPV+ cervical cancer [162]. Together, these activities lead to uncontrolled oncogenic YAP1 activity, contributing to the increased proliferation and survival necessary for tumourigenesis.

#### JAK/STAT signalling pathway

The Janus kinase/signal transducer and activator of transcription (JAK/STAT) pathway is a key signalling axis associated with the regulation of embryonic development, stem-cell maintenance and the inflammatory response. Further, dysregulation of JAK/STAT signalling has also been shown to promote cancer progression, immune evasion and metastasis [163]. Pathway activation occurs upon binding of cytokines, interleukins and growth factors to a number of transmembrane receptors. This in turn results in activation of JAKs, followed by the recruitment, phosphorylation and activation of STAT proteins. Subsequently, STAT dimers translocate to the nucleus and bind to specific promoter sequences, leading to the transcription of target genes.

STAT3 drives proliferation in stratified epithelia and is essential for the maintenance of stemness. Studies in our lab have demonstrated that STAT3 phosphorylation is increased in HPV-containing keratinocytes in an E6-dependent manner and that this is required for episome maintenance in undifferentiated cells as well as the continued proliferation of suprabasal cells [20]. A number of studies have confirmed that STAT3 phosphorylation is also increased in cervical cancer [164]; this is achieved through increased NFκB signalling and autocrine/paracrine activation of STAT3 by the pro-inflammatory cytokine interleukin-6 (IL-6) [21]. Importantly, we have shown that STAT3 activity, as well as that of the upstream kinase JAK2, is essential for proliferation and survival of cervical cancer cells [21, 165].

### E6-mediated immune evasion

Evidence also suggests that E6 is capable of modulating the immune response to infection. HR-HPV E6 proteins interact with a component of the innate immune response: IFN regulatory factor 3 (IRF3) [166]. This interaction is thought to prevent the transactivation of IFNβ expression and hence the induction of ISGs. Furthermore, HPV18 E6 has been demonstrated to directly interact with and impair activation of the non-receptor tyrosine kinase 2 (TYK2), thus inhibiting the induction of JAK/STAT signalling [167]. Disruption of the immune response can also be achieved via dysregulation of the RIG-I pathway: E6 proteins from several high-risk types bind to TRIM25 and USP15, two key activators of RIG-I, promoting the ubiquitination and degradation of TRIM25 and ultimately inhibiting the activation of RIG-I signalling [168]. Furthermore, E6 inhibits transcription of the keratinocyte-specific IFNκ, in addition to E5-induced methylation of its promoter, to prevent activation of antiviral ISGs and pattern recognition receptors (PRRs) [97, 169].

### Epigenetic reprogramming of host cells

Significant alterations to host chromatin modification patterns, including DNA methylation and histone acetylation/methylation, are observed during HPV infection. Aberrant DNA methylation at promoter regions is an important way in which loss of tumour suppressor gene expression can occur during carcinogenesis and has been reported in cervical cancer [170]. One of the major mechanisms associated with this likely involves oncoprotein activation of DNMT1, the methyltransferase responsible for maintenance of DNA methylation levels. Indeed, HPV infection has profound effects upon the host methylome: 8000 and 10 000 genes were demonstrated to have altered DNA methylation levels, either increased or decreased, in primary keratinocytes harbouring episomal HPV16 and HPV18 genomes, respectively [171]. E6 indirectly increases DNMT1 expression through the degradation of p53 [172]. p53 negatively regulates DNMT1 expression by binding in concert with Sp1 to the *DNMT1* promoter but in the absence of p53, Sp1 acts to enhance DNMT1 expression [173]. Whilst the full functional impact of E6-mediated modulation of the host methylome remains to be understood, it has been shown that E6 expression leads to hypermethylation and hence silencing of the *death associated protein kinase 1 (DAPK1*) promoter, a tumour suppressor that is a key mediator of IFNγ-induced, as well as apoptotic, cell death [174].

Additionally, E6 can affect host chromatin structure via the dysregulation of histone-modifying enzymes. Both high-risk and low-risk E6 proteins interact with the histone methyltransferases coactivator associated arginine methyltransferase 1 (CARM1), protein arginine methyltransferase 1 (PRMT1) and SET7, resulting in inhibition of their enzymatic activity [175]. Together, this provides another mechanism by which HPV modulates p53 function: CARM1 and PRMT1 are required to activate transcription from p53-responsive promoters, whilst SET7 has an additional function in methylating, and hence stabilizing, p53 [175]. Importantly, however, loss of the activity of these methyltransferases is likely to have wider impacts upon cellular functions due to global changes in gene transcription, which remain to be elucidated.

### Targeting of non-coding RNAs

Emerging evidence indicates that the HPV oncoproteins are also capable of targeting non-coding RNA (ncRNA) species, including microRNAs (miRNAs), long non-coding RNAs (lncRNAs) and circular RNAs (circRNAs), in order to manipulate the host-cell environment. All three of these ncRNA types have been documented to be dysregulated in HPV infection and cervical cancer, with effects that promote proliferation and contribute to delayed differentiation (see [176] for a review).

miRNAs are short, ~22 nucleotide non-coding RNA species expressed in nearly all eukaryotes [177]. miRNAs typically downregulate gene expression by binding to the 3′UTR of target transcripts, promoting their cleavage. Despite the numerous studies evaluating the expression of miRNAs in cervical neoplasia and cervical cancer tissue biopsies, the mechanisms behind the observed changes in expression are often poorly understood. However, HR-HPV E6 has been demonstrated to directly regulate the expression of a collection of miRNAs. Among these, downregulation of the tumour suppressive miRNAs miR-34a and miR-218 has been reported in both HPV+ cell lines and HPV+ cervical lesions [178, 179]. Conversely, E6-induced enhancement of miR-20a and miR-20b expression likely contributes towards increased proliferation and invasion of cervical cancer cells [180, 181]. Furthermore, studies in our group indicate that oncoprotein-induced expression of the oncogenic miR-18a contributes towards the dysregulation of Hippo signalling discussed previously by silencing STK4 expression [162]. Importantly, we demonstrated that reintroduction of STK4, or transfection of an antagomir to inhibit miR-18a, led to a reduction in proliferation [162].

LncRNAs are RNA molecules greater than 200 nucleotides long and possess many of the same structural features of mRNAs but lack a recognizable ORF [182]. They can localize to both the nuclear and cytoplasmic compartments of the cell and have many modes of action, including inhibition of miRNAs via sponging, epigenetic regulation of gene expression and stabilization of mRNA transcripts [182]. LncRNAs have been documented to promote cancer development through their regulation of proliferation, apoptosis and invasion, and could also serve as cancer biomarkers. Cervical cancer is no exception to this, and a number of lncRNAs have been demonstrated to be dysregulated during progression [176]. For example, expression of the cervical carcinoma expressed PCNA regulatory (CCEPR) lncRNA is markedly increased in cervical cancer, and expression directly correlates with tumour size and poor prognosis [183]. Expression of CCEPR was later shown to be driven by HPV16 E6 in a p53 degradation-independent manner [184]. The expression of other lncRNAs, including FAM83H antisense RNA 1 (FAM83H-AS1) and H19, was also demonstrated to be increased in HPV16 E6-expressing primary keratinocytes, although precise mechanisms are yet to be elucidated [185].

## The E7 oncoprotein

**Fig. 5.**
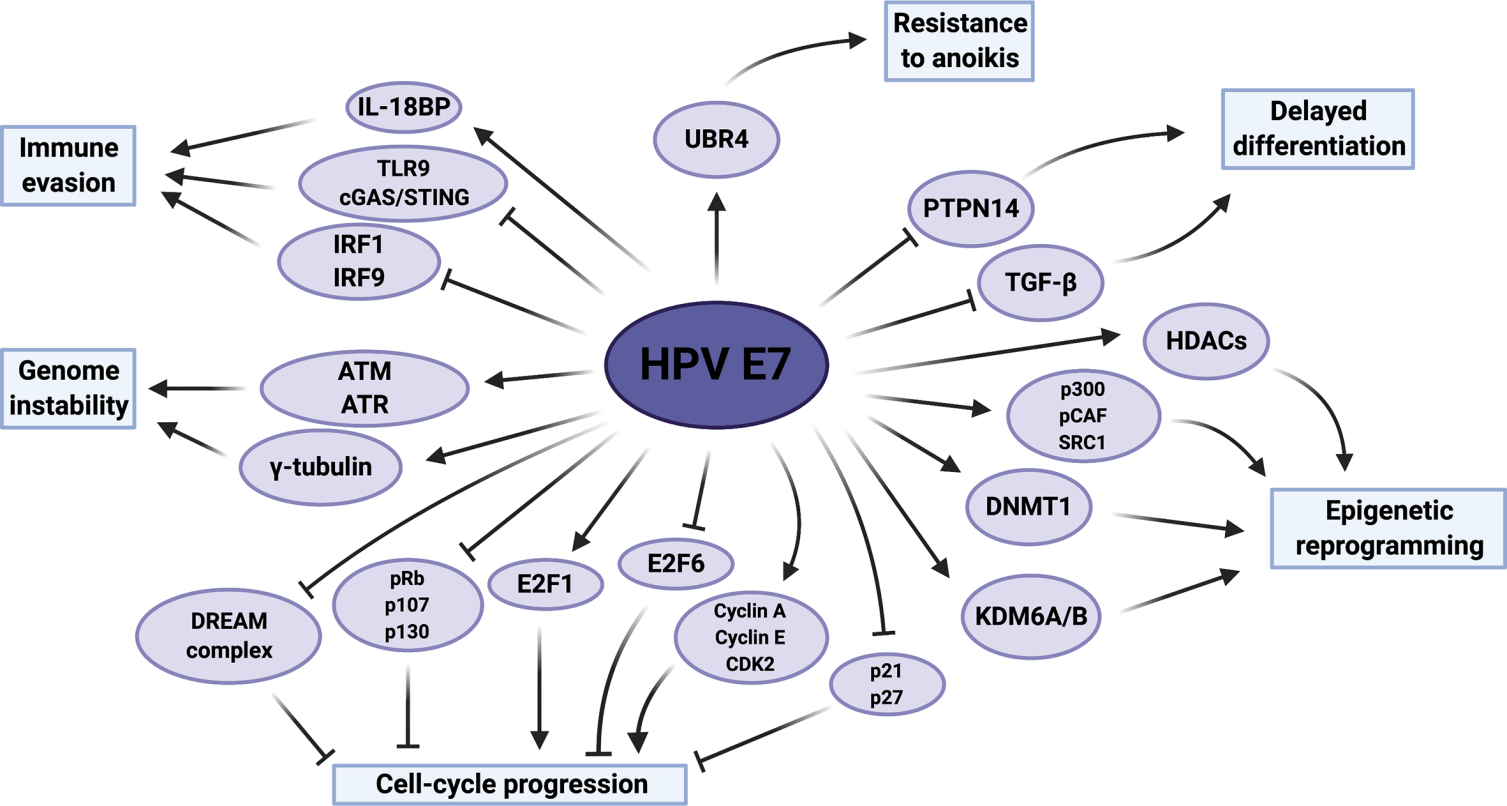
Summary of HPV E7 activities which contribute towards host-cell transformation. The host proteins and pathways reported to be modulated by HPV E7 are displayed, as well as the functional impact these have on the host cell. PTPN14, protein tyrosine phosphatase non-receptor type 14; TGFβ, transforming growth factor beta; HDACs, histone deacetylases; SRC1, steroid receptor coactivator 1; DNMT1, DNA methyltransferase 1; KDM6A/B, lysine demethylase 6A/B; CDK2, cyclin-dependent kinase 2; pRb, retinoblastoma protein; DREAM, dimerization partner, pRb-like, E2F and multi-vulval class B; ATM, ataxia-telangiectasia mutated; ATR, ataxia-telangiectasia and Rad3-related; IRF, IFN regulatory factor; TLR9, Toll-like receptor 9; cGAS, cyclic GMP-AMP synthase; STING, stimulator of IFN genes; IL-18BP, interleukin-18 binding protein. Figure created using BioRENDER.com.

The E7 protein is considered to be the major transforming protein of HR-HPVs and its continued expression is essential for carcinogenesis [186, 187]. Underlining its importance, sequencing of viral genomes has revealed remarkable amino acid sequence conservation of the E7 protein in high-grade cervical neoplasia and cervical cancer specimens [188]. The protein is ~100 amino acids long and possesses two conserved regions (CR1 and CR2) at its N-terminus, which show significant similarity to other DNA virus oncoproteins, including adenovirus E1A and simian virus 40 (SV40) large tumour antigen (Fig. 1c) [189]. Within CR2 lies the conserved LXCXE motif necessary and sufficient for binding to pRb, and a casein kinase II phosphorylation site [12, 190, 191]. The C-terminus of E7 is less well-conserved except for two CXXC zinc-binding motifs [186]. E7 possesses both nuclear localization and nuclear export signals (NLS and NES, respectively), suggesting that it shuttles between the cytoplasm and nucleus and has functions in both compartments [192]. Much like E6, the E7 oncoprotein is multifunctional (Fig. 5), and is reported to interact with numerous host proteins (Table 3).

**Table 3.** Host proteins known to interact with HPV E7

Protein name	HR-HPV	LR-HPV	Proposed function	Reference
Actin (F-actin)	+	?	Cytoskeletal rearrangements	[316]
AP-1 (c-Jun, JunB, JunD, c-Fos isoforms)	+	?	Increased transforming ability	[317]
ATM	+	?	DDR activation	[233]
B-Myb/MuvB complex	+	low	Increased proliferation	[318]
BRCA1	+	?	Defective DNA repair	[283]
BRG1	+	-	Increased proliferation	[319]
Calpain	+	?	pRb destabilization	[200]
Casein kinase II	+	+	Increased transforming ability	[190, 191]
CDK2	+	+	Increased proliferation	[207, 320]
CENP-C	+	-	Unknown	[321]
CHD4	+	?	Increased proliferation	[250]
c-Myc	+	+	Increased c-Myc activity	[322]
Cullin 1	+	?	Ubiquitin-mediated degradation of E7	[323]
Cullin 2	HPV16 only	-	pRb degradation	[198, 199]
Cullin 3	+	+	Unknown	[199]
Cyclin A	+	+	Increased proliferation	[14, 207]
Cyclin E	+	+	Increased proliferation	[207, 320]
DNMT1	+	?	Altered host methylome	[246]
DYRK1A	+	?	Phosphorylation and stabilization of E7	[324]
E2F1	+	low	Increased proliferation	[203]
E2F6	+	+	Increased proliferation	[206]
ENC1	HPV18/45 only	-	Unknown	[199]
FHL2	+	?	Impaired FHL2 coactivator function	[325]
FOXM1	+	?	Increased transforming ability	[326]
Gelsolin	+	?	Cytoskeletal rearrangements	[327]
GRP78	+	?	Increased oncoprotein stability	[328]
GSTP1	+	?	Increased survival	[329]
HDAC1	+	?	Increased proliferation	[250]
HDAC2	+	?	Increased proliferation	[250]
HIF-1α	+	+	Promotes angiogenesis	[330]
HTRA1	+	?	Unknown	[331]
IGFBP-3	+	low	Increased survival	[332]
IKKα	+	?	Immune evasion	[323]
IKKβ	+	?	Immune evasion	[333]
IRF1	+	+	Immune evasion	[226]
IRF9	+	?	Immune evasion	[225]
KCMF1	+	+	Unknown	[199]
MAP4	HPV16 only	low	Deregulation of mitosis	[334]
Miz-1	+	+	Inhibition of p21 expression	[335]
NDKA	+	?	Unknown	[336]
NDKB	+	?	Unknown	[336]
NLRX1	HPV16 only	?	Immune evasion	[337]
NuMA1	+	+	Deregulation of mitosis	[338]
Nup62	+	?	Nuclear import of E7	[339]
Oct4	+	?	Unknown	[340]
p107	+	low	Increased proliferation	[14, 202, 341]
p130	+	low	Increased proliferation	[14, 202, 341]
p190RhoGAP	+	+	Cytoskeletal rearrangements	[342]
p21^CIP1^	+	low	Increased proliferation	[210, 211]
P26s4	+	?	Increased proteasome activity	[343]
p27^KIP1^	+	low	Increased proliferation	[209]
p300	+	low	Epigenetic reprogramming	[255]
pCAF	+	+	Epigenetic reprogramming	[253, 254]
PKM2	+	-	Metabolic changes	[344]
PML	+	+	Inhibition of PML-induced senescence	[345]
PP2A	+	+	Increased PI3K/Akt signalling	[346]
pRb	+	low	Increased proliferation	[12, 13, 202]
PTPN14	+	+	Increased proliferation; impaired differentiation	[212–215]
PTPN21	+	?	Unknown	[215]
Ran	+	?	Nuclear import of E7	[347, 348]
Siva	+	?	Increased survival	[349]
SMAD1	+	+	Increased proliferation	[350]
SMAD2	+	+	Increased proliferation	[217, 350]
SMAD3	+	+	Increased proliferation	[217, 350]
SMAD4	+	+	Increased proliferation	[217, 350]
SNW1	+	low	Inhibition of SNW1 coactivator function	[351]
SRC1	+	-	Epigenetic reprogramming	[256]
STING	HPV18 only	?	Immune evasion	[219, 337]
TAF1C	+	?	Dysregulation of RNA polymerase I transcription	[352]
TAP1	+	?	Immune evasion	[353]
TBP	+	?	Inhibition of TBP DNA binding	[354]
TG2	+	+	Unknown	[355]
UBR4	+	+	Resistance to anoikis; PTPN14 degradation	[199, 212, 245]
ZER1	HPV16 only	-	pRb degradation	[199]
α-glucosidase	+	?	Increased glycogen turnover	[356]
γ-tubulin	+	?	Defective centrosome duplication	[241]

+, interaction confirmed; -, no interaction recorded; low, limited binding detected; ?, binding ability not analysed.

### Targeting of the retinoblastoma protein (pRb) family of tumour suppressors

The best-characterized function of E7 is dysregulating the G1/S-phase transition in order to promote increased proliferation by disrupting E2F transcription factor activity. The E2F transcription factors typically consist of an E2F subunit (E2F1-6) and a DP subunit (DP1-3), although two additional non-canonical family members, E2F7 and E2F8, have been reported (see [193] for a review). E2F transcription factors can be broadly divided into activators of transcription (E2F1, 2, 3a and 3b), which preferentially bind pRb, and repressors (E2F4-8). Within a naïve cell, phosphorylation of pRb by CDK2, CDK4 or CDK6 during G1 phase induces dissociation of E2Fs, leading to expression of genes necessary for S-phase progression. However, during infection, dysregulation of E2F transcription factors is achieved primarily by binding of E7 to pRb and the related pocket proteins p107 and p130 via the conserved LXCXE motif located within its CR2 domain [12–14]. Some evidence indicates that sequences within the C-terminus of E7 may also be required for pRb binding [194]. Binding of E7 disrupts pRb-E2F complexes, releasing E2Fs from repression and hence enabling the expression of E2F-dependent genes associated with cell-cycle progression such as cyclins A and E, contributing towards S-phase re-entry in differentiating suprabasal keratinocytes [11]. Concurrently, binding of HPV E7 to p107 and p130 leads to disruption of the dimerization partner, pRb-like, E2F and multi-vulval class B (DREAM) repressor complex, permitting a further enhancement in the expression of proliferative genes [195]. E7-pocket protein binding and S-phase entry is significantly enhanced by casein kinase II phosphorylation of serine-32 and -34 residues within the CR2 domain [196, 197].

Further, HR-HPV E7 proteins can target Rb family members for proteasomal degradation via a mechanism that requires binding to the cullin 2 ubiquitin ligase complex by E7 [15, 16, 198, 199]. E7-induced degradation also requires cleavage of pRb by the protease calpain-1; this unmasks a C-terminal unstructured region of pRb, which acts as an efficient initiation region for degradation by the proteasome [200, 201]. In contrast, low-risk E7 proteins have a significantly weaker affinity for the pocket proteins, which likely contributes to their lack of transforming activity [12]. Interestingly, only p130 degradation has been reported for the low-risk E7 proteins, suggesting that destabilization of this pocket protein is vital for the HPV life cycle yet dispensable for tumourigenesis [202].

### Other mechanisms underlying dysregulation of the G1/S cell-cycle checkpoint

In addition to indirectly modulating E2F activity through targeting pRb, E7 also possesses other mechanisms of affecting E2F transcription factors. A direct interaction between E7 and E2F1 has been reported; this leads to a further increase in E2F-dependent gene expression [203]. A consequence of this is heightened expression of the repressive E2F6 transcription factor, which likely constitutes a feedback loop to prevent inappropriate expression of E2F1-dependent genes at other stages of the cell cycle [204]. E2F6 acts as a transcriptional repressor of E2F-responsive promoters by recruiting polycomb group (PcG) complexes and histone methyltransferases [205]. This negative feedback loop can be negated by binding of E7 to E2F6, leading to its inactivation [206].

**Fig. 6.**
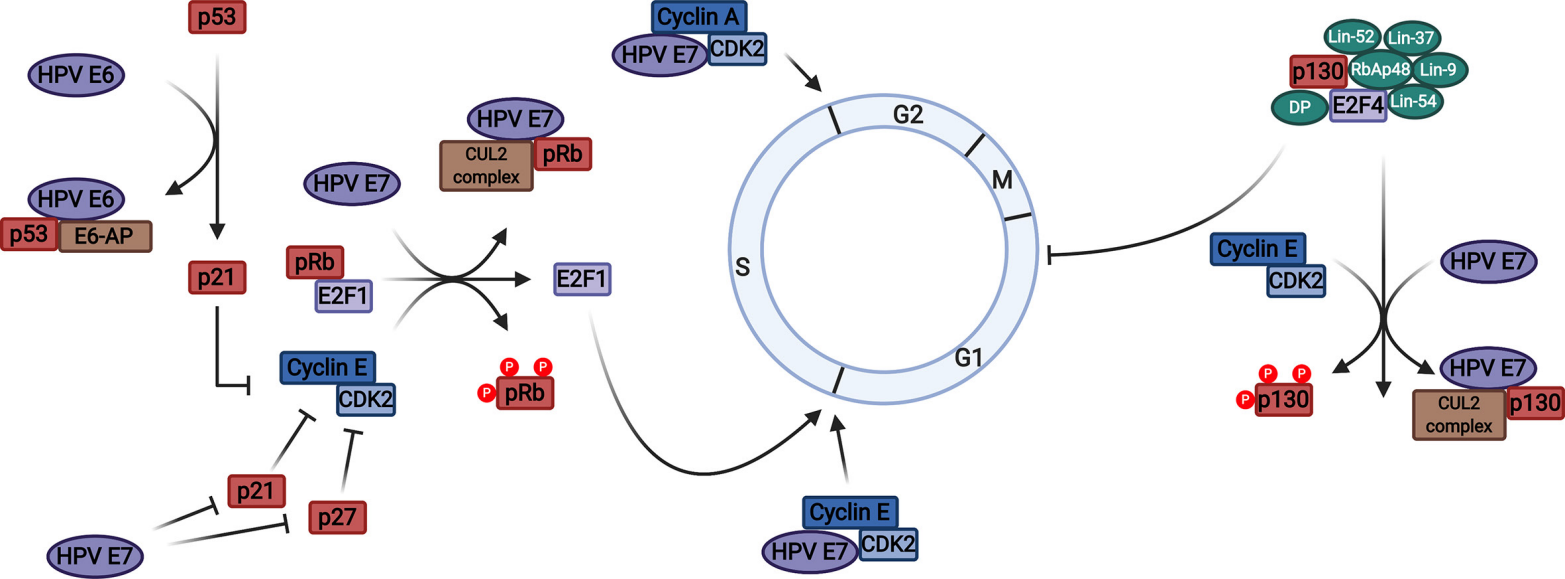
Dysregulation of cell-cycle checkpoints by HPV E7. The mechanisms employed by HPV to include cell-cycle progression are illustrated. HPV E7 binds to and induces the degradation of the retinoblastoma protein (pRb) in a cullin 2 (CUL2) ubiquitin ligase-dependent manner. This releases the E2F1 transcription factor from inhibitory complexes, permitting the expression of genes associated with S-phase progression. Degradation of the related pocket proteins p107 and p130 prevents inhibition of G1 progression by the DREAM complex. HPV E7 also binds directly to cyclins A and E, potentiating cyclin-dependent kinase 2 (CDK2) activity. Further, HPV E7 can suppress the activity of the CDK inhibitors p21 and p27, allowing increased cyclin/CDK phosphorylation of pRb and enhanced E2F1-dependent transcription. HPV E6 also indirectly contributes towards cell-cycle progression: E6-associated protein (E6-AP)-dependent degradation of p53 prevents induction of p21 expression. Figure created using BioRENDER.com.

E7 proteins are also able to dysregulate the G1/S-phase checkpoint in other ways. The activity of CDK2, a kinase crucial for S-phase entry and progression, is maintained via an interaction between its cognate cyclins (cyclin E and cyclin A) and E7 (Fig. 6) [207]. This is on top of already heightened cyclin A and E levels; their expression is regulated by E2Fs and is hence increased in E7-containing cells [208]. Further, the activities of the CDK inhibitors p21^CIP1^ and p27^KIP1^, which are implicated in mediating keratinocyte differentiation via inducing a G1 cell-cycle arrest, are suppressed by E7 binding (Fig. 6) [209–211]. Together these mechanisms act to ensure the efficient S-phase re-entry of differentiating suprabasal epithelial cells.

### Impairment of differentiation

In addition to its roles in driving proliferation, emerging evidence indicates that E7 may also play a role in impairing cellular differentiation. A diverse range of E7 proteins possess the ability to bind the non-receptor protein tyrosine phosphatase PTPN14, with HR-HPV E7 proteins additionally able to target it for proteasomal degradation using the UBR4 ubiquitin ligase [212, 213]. This loss of PTPN14 is critical in both delaying the epithelial differentiation programme and for cellular transformation, although evidence that this is achieved via modulation of Hippo pathway signalling remains controversial [214, 215]. Interestingly, mutational analyses indicate that this constitutes one of the as yet poorly defined pRb-independent functions of E7: regions in both CR1 and the C-terminus are necessary for formation of the E7/PTPN14/UBR4 complex.

Manipulation of the TGFβ pathway by E7 may also contribute towards delaying epithelial differentiation. Expression of HPV16 E7 was shown to block TGFβ-mediated suppression of c-Myc expression [216]. Mechanistically, one report claimed that the negative effects of TGFβ signalling on cell proliferation are abrogated by binding of HPV16 E7 to SMAD2, SMAD3 and SMAD4, thus preventing the association of SMADs with their DNA-binding elements [217].

### E7-mediated immune evasion

Much like the other oncoproteins, E7 plays a role in immune evasion by HPV by targeting several stages of the cellular antiviral response. Indeed, recent evidence suggests that expression of both E6 and E7 is necessary to repress the innate immune response [218]. A key target for HPV are the host PRRs: direct binding of HPV18 E7 to STING, in a manner dependent on its LXCXE pRb-binding motif, results in inhibition of the cGAS-STING signalling axis, a major pathway involved in the recognition of exogenous DNA [219]. Signalling is further repressed in HPV+ cervical cancer cells via E7-induced upregulation of the H3K9-specific DNA methyltransferase SUV39H1, leading to the suppression of cGAS and STING expression [220]. The histone demethylases KDM5B and KDM5C also participate in *STING* silencing by promoting removal of the activatory histone marker H3K4me3 [221]. Indeed, expression of KDM5B and STING are negatively correlated in HPV+ cancers [221]. Additionally, HPV16 E7 is able to promote the epigenetic silencing of the dsDNA sensor Toll-like receptor 9 (TLR9) via recruitment of KDM5B and the histone deacetylase HDAC1 to the *TLR9* promoter [222]. Together, these measures act to prevent production of type I IFNs.

Immune signalling downstream of PRRs is also modulated by HPV. The transcription factor NFκB can be activated by numerous inflammatory stimuli and plays a major role in anti-viral immunity. Our group identified that E7 proteins from both high-risk and low-risk types prevented activation of NFκB signalling in primary keratinocytes, leading to reduced secretion of pro-inflammatory cytokines [223]. Further, we showed that E7 expression augments secretion of IL-18 binding protein (IL-18BP), an anti-inflammatory cytokine, in response to IFNγ stimulation [224].

The IFN response is also suppressed via binding of E7 to IRF9, abrogating the expression of ISGs [225]. IRF1 is similarly thought to be targeted by high-risk E7 proteins: direct binding of HPV16 E7 to the C-terminus of IRF1 precludes binding to DNA and hence prevents expression of target genes such as IFNβ [226].

Furthermore, mouse models have demonstrated that HPV16 E7 expression results in a locally immunosuppressive environment where cytotoxic T lymphocyte function is significantly suppressed [227]. Mechanistically, this could be associated with reduced expression of the chemokine CXCL14, observed in both cervical cancer and HPV+ HNSCC tissue, due to E7-dependent promoter hypermethylation. Importantly, reintroduction of CXCL14 results in increased immune cell infiltration and suppresses tumour growth [228]. HPV E7 may also interfere with MHC class I surface expression, in addition to the role played by E5, in order to prevent NK cell-mediated killing [229].

### Replication stress and genomic instability

Genomic instability is a hallmark of HPV+ cancers, but is also frequently observed in premalignant cervical lesions [230]. Aberrant activation of the pRb-E2F pathway by HPV E7 and the ensuing unscheduled proliferation is sufficient to induce replication stress and genomic instability due to inadequate nucleotide pools [231, 232]. In support of this, HPV has been demonstrated to activate both the ATM (ataxia-telangiectasia mutated) and ATR (ataxia-telangiectasia and Rad3-related) DNA damage response (DDR) pathways, which are induced in response to DNA double-strand breaks and replication stress respectively [233–235]. Significantly, inhibition of either of these DDR signalling pathways has an adverse effect on viral genome amplification, suggesting that the virus has evolved to not only tolerate replication stress and the DDR, but to require them for its life cycle [233, 234]. Mechanistically, ATM activation is thought to be accomplished via a non-canonical route involving STAT5 signalling, which is at least in part E7-driven, and the acetyltransferase Tip60 [236, 237]. STAT5 is also implicated in driving the ATR-DDR response: HPV31 E7-induced STAT5 phosphorylation drives transcription of TopBP1, which subsequently binds and stimulates the kinase activity of ATR [234]. Importantly, the E1 helicase can similarly activate the DDR, and a recent study demonstrated that DDR activation occurs to the same extent in keratinocytes containing HPV16 genomes lacking functional E6 and E7 ORFs as with wild-type HPV16 genomes, leading to the hypothesis amongst some that viral replication itself may also play a key role in DDR activation [218].

Intriguingly, a recent study suggests that DNA repair factors may be recruited away from host DNA towards viral genomes [232]. Indeed, several ATM-DDR signalling components, including ATM, γH2AX, Chk2 and BRCA1, are localized to the sites of viral replication during an infection, perhaps to ensure high-fidelity genome replication [238]. This has led to the hypothesis that HPV episomes are shielded from the DNA damage ensuing from oncoprotein-induced replication stress, and that this may contribute to the DNA lesions and genetic instability in the host genome necessary for progression to cancer [232].

Other potential methods of inducing genomic instability have also been reported. Expression of HPV16 E7 can rapidly induce abnormal centrosome numbers in the host cell due to defective centrosome duplication, which in turn permits chromosome missegregation, aneuploidy and genomic instability [239, 240]. Additionally, an association between E7 and γ-tubulin has been reported [241]. This purportedly delays the recruitment of γ-tubulin to centrosomes, further contributing to the subversion of centrosome duplication.

### Resistance to anoikis

Anoikis is a form of programmed cell death induced upon detachment from the extracellular matrix and resistance to anoikis (i.e. anchorage-independent growth) is a key hallmark of cancer cells [242]. Expression of BPV1 E7 was found to stimulate anchorage-independent cell growth and this correlated with its ability to bind UBR4 (also known as p600) [243, 244]. An interaction between UBR4 and HPV16 E7 was also reported and indeed this ability to bind UBR4 was later found to be conserved across many HPV types [199, 245]. Importantly, depletion of UBR4 in the presence of E7 inhibited anchorage-independent growth, suggesting this is likely a mechanism by which papillomaviruses dysregulate anoikis [243, 245]. More recently, the interaction between E7 and UBR4 was found to be required for PTPN14 degradation and that this contributes towards delaying keratinocyte differentiation, as discussed previously in this section [212–214]. However, PTPN14 knockout also reduced detachment-induced cell death, perhaps indicating that targeting of this pathway by HPV may concurrently impact upon differentiation and anoikis [214].

### Epigenetic reprogramming of host cells

In addition to the role played by E6 in dysregulating host epigenetic regulatory mechanisms, E7 can also induce significant alterations to cellular chromatin modification patterns. In some cases, the oncoproteins concurrently target the same host factor, such as the DNA methyltransferase DNMT1. Further to indirect activation of DNMT1 through E6-mediated p53 degradation, direct binding of HPV16 E7 to DNMT1 is sufficient to promote its enzymatic activity [246]. Moreover, the expression of DNMT1 and DNMT3B is reportedly increased in keratinocytes harbouring episomal HPV genomes [171].

E7 can additionally induce changes to cellular histone modifications. As discussed elsewhere in this review, modulating the expression and activity of histone-modifying enzymes can contribute to suppression of the host immune response [220–222]. In addition to this, a genome-wide reduction in levels of the repressive histone mark H3K27me3 has been reported in HPV16 E7-expressing keratinocytes due to transcriptional induction of the KDM6A and KDM6B lysine demethylases, whilst KDM6A expression is also significantly upregulated in HPV+ tumours [247, 248]. Cervical cancer cells have been demonstrated to be addicted to KDM6A: it is thought that loss of H3K27 trimethylation due to KDM6A activity promotes p21^CIP1^ expression, which is required to survive E7-induced replication stress [249] .

E7 also interacts with histone deacetylases (HDACs), which function by removing acetyl groups from histone lysine residues, thus inducing chromatin remodelling and transcriptional repression [250]. Binding of E7 to HDACs was found to be essential for the HPV31 life cycle; this is likely associated with promoting S-phase progression in differentiating keratinocytes by increasing levels of E2F2-dependent transcription [251, 252]. Similarly, E7 can also associate with the histone acetyltransferases (HATs) p300, pCAF and SRC1 [253–256]. Whilst the biological relevance remains to be fully elucidated, it is clear that these interactions can result in reduced HAT activity.

### Targeting of non-coding RNAs

Like E6, recent evidence indicates that HPV E7 can directly modulate the expression of multiple miRNAs, such as miR-203. The primary role of miR-203 is to promote epithelial differentiation by inducing cell-cycle exit, and as such levels of miR-203 increase in a differentiation-dependent manner [257]. Induction of the differentiation programme is achieved by downregulating expression of p63, a key driver of proliferation in epithelial tissue, which is commonly overexpressed in both HNSCC and cervical cancer [257, 258]. Suppression of miR-203 expression by E7 permits increased protein levels of ΔNp63, the major epithelial p63 isoform, and hence contributes to delaying epithelial differentiation [258]. Other miRNAs purportedly targeted by E7 include miR-16, miR34b, miR-34c-5p and miR-486–5 p, although the significance of these in relation to the HPV life cycle and cancer progression, as well as exact mechanisms, remain to be elucidated [259].

Co-regulation of miRNAs by E6 and E7 has also been reported: both oncoproteins contribute to the suppression of miR-424 in order to promote HPV genome amplification, whilst our data indicates that upregulation of the oncogenic miR-18a is necessary for transformation, as discussed previously [162, 260].

The expression of a number of lncRNAs can also be modulated by E7. Contrasting reports exist on the effect of E7 on expression of the HOX transcript antisense intergenic RNA (HOTAIR): one study indicated that HOTAIR was highly expressed in cervical cancer tissue and cell lines [261], whilst others suggest HOTAIR is in fact downregulated [262, 263]. A physical interaction between HPV16 E7 and HOTAIR has also been reported [262]. This is hypothesized to disrupt normal HOTAIR function by impeding its binding to the Polycomb repressive complex 2 (PRC2) and the histone lysine demethylase KDM1A, ultimately leading to the derepression of target genes. Other lncRNAs reportedly regulated by E7 include LINC01101 and LINC00277, both of which demonstrate low expression levels in cervical cancer specimens and are upregulated after siRNA silencing of E7 [264].

Interestingly, E7 may also act cooperatively with E6 to modulate lncRNA activity. Expression of thymopoietin pseudogene 2 (TMPOP2) was found to be upregulated in an oncoprotein-dependent manner in HPV+ cervical cancer cells as well as in HPV16 E6/E7 expressing keratinocytes [263, 265]. Depletion of TMPOP2 resulted in a decrease in proliferation due to arrest in G1 phase and a reduction in cyclin E and CDK2 expression [265]. Further, the oncoproteins have also been implicated in upregulating expression of the widely-studied metastasis associated lung adenocarcinoma transcript 1 (MALAT1) lncRNA [266]. Dysregulation of lncRNAs by HPV may also contribute towards delaying the onset of differentiation in keratinocytes: expression of HPV16 E6 and E7 in primary keratinocytes resulted in increased expression of differentiation antagonizing non-protein coding RNA (DANCR), whilst tissue differentiation-inducing non-protein coding RNA (TINCR) expression was suppressed [263]. High DANCR expression has similarly been reported in cervical cancer tissue, and depletion of DANCR significantly inhibited proliferation, migration and invasion, whilst siRNA knockdown of TINCR promoted cell growth [267, 268].

Finally, emerging evidence indicates that E7 may also dysregulate the expression of circRNAs. These are a collection of covalently closed ncRNAs produced by a unique splicing mechanism and function most commonly as sponges for miRNAs and in regulating gene transcription [269]. Microarray analysis demonstrated that upon E7 knockdown in CaSki cells, over 500 circRNAs were found to be altered in expression level [270]. However, the functional significance of this is yet to be understood. Other studies have also identified differentially expressed circRNAs in cervical cancer tissue, but whilst they have been linked to proliferation and cancer progression, it is as yet unclear whether changes in their expression are a direct result of oncoprotein activity [271–273]. Interestingly, HPV16 has been demonstrated to express its own circRNAs, one of which contains the entire E7 ORF and is hence termed circE7 [274]. Somewhat surprisingly, rather than functioning as a miRNA sponge, circE7 was found to be required for optimal E7 protein expression and circE7 depletion significantly impeded the proliferation of HPV+ cervical cancer cells [274].

## Conclusions

The HPV oncoproteins E5, E6 and E7 have evolved to hijack many host signalling pathways in order to generate a cellular environment conducive for virus replication. This includes the widely studied functions of E6 and E7 in targeting p53 and pRb, respectively, for proteasomal degradation. These, together with the other oncoprotein functions discussed in this review, act to stimulate proliferation, delay differentiation, inhibit apoptosis and evade immune detection. An undesired consequence of this is the acquisition of mutations in cellular genes, particularly in the context of dysregulated oncoprotein expression, which can facilitate transformation. Although much progress has been made in understanding the host pathways modulated by HPV, novel mechanisms continue to be elucidated. Gaining a thorough understanding of the cellular proteins and pathways subverted by HPV during infection, and in particular during carcinogenesis, will aid in the development of novel therapeutic agents.
